# G-protein coupled receptor 34 regulates the proliferation and growth of LS174T cells through differential expression of PI3K subunits and PTEN

**DOI:** 10.1007/s11033-021-07068-4

**Published:** 2022-01-08

**Authors:** Bo Zuo, Na Wu, Shen Yang, Zhaohui Zhong, Mei Li, Xin Yu, Yulan Liu, Weidong Yu

**Affiliations:** 1 Department of Central Laboratory & Institute of Clinical Molecular Biology, Peking University People’s Hospital, Beijing, 100044 People’s Republic of China; 2 Department of General Surgery, Peking University People’s Hospital, Beijing, 100044 People’s Republic of China; 3 Department of Hepatobiliary Surgery, Peking University People’s Hospital, Beijing, 100044 People’s Republic of China; 4 Department of Gastroenterology, Peking University People’s Hospital, Beijing, 100044 People’s Republic of China

**Keywords:** GPR34, Proliferation, Xenograft tumor growth, PI3K subunits, Colorectal cancer

## Abstract

**Purpose:**

G-protein coupled receptor (GPR 34) has been found to play important roles in some cancers and regulates the proliferation, apoptosis, and migration of these cancer cells. However, the mechanisms underlying how GPR34 functions to regulate growth and proliferation of colorectal cancer cells remains to be clarified.

**Methods:**

We employed stable GPR34 knockdown LS174T cell models, GPR34 Mab blocking, a CCK-8 kit, and a colony formation assay to characterize the effect of GPR34 on the proliferation of LS174T in vitro and xenograft tumor growth in vivo. The mRNA level of GPR34 was detected by RT-PCR in tumor tissues and adjacent normal tissues from 34 CRC patients.

**Results:**

Based on RT-PCR results, GPR34 exhibited high level in tumor samples compared with adjacent normal samples. Increased expression of GPR34 is more associated with poor prognosis of CRC as shown in The Cancer Genome Atlas (TCGA) dataset by Kaplan–Meier survival analysis. Furthermore, we showed that GPR34 knockdown inhibited the proliferation of LS174T colon cancer cells and related xenograft tumor growth. Searching for the distinct molecular mechanism, we identified several contributors to proliferation of LS174T colon cancer cells: PI3K subunits/PTEN, PDK1/AKT, and Src/Raf/Ras/ERK. GPR34 knockdown inhibited the proliferation of LS174T cells by upregulating expression of PTEN, and downregulating expression of PI3K subunits p110-beta.

**Conclusion:**

Our findings provide direct evidence that GPR34 regulates the proliferation of LS174T cells and the growth of LS174T tumor xenografts by regulating different pathways. High expression of *GPR34* mRNA could then be used to predict poor prognosis of CRC.

**Supplementary Information:**

The online version contains supplementary material available at 10.1007/s11033-021-07068-4.

## Introduction

G-protein coupled receptor 34 (GPR34) is a 7-transmembrane receptor that regulates key biological functions including cellular growth, motility, apoptosis, and gene transcription, and also appears to be involved in the progression of several cancers [1–9]. Several studies have addressed elevated expression levels of GPR34 in malignancies, including cervical cancer [7], metastatic melanoma [6], MALT lymphoma [1, 2], BCR/ABL-positive leukemia [9], gastric cancer [5, 8], and colon cancer [4], as well as its essential roles in tumor development and progression. Overexpression of GPR34 in lymphoma and HeLa cells results in phosphorylation of ERK, PKC, and CREB; induces CRE, AP1, and NF-κB-mediated gene transcription; and increases cell proliferation [1]. Our previous studies have demonstrated that increased expression of GPR34 was involved in the proliferation of gastric cancer HGC-27 cells via the PI3K/AKT pathway [5]. Then, we identified GPR34/PI3K/AKT as an alternative pathway that may mediate p185Bcr/Abl-induced transformation and leukemogenesis. Interestingly, these studies also indicated that GPR34 plays its pro-proliferation role by effecting different PI3K subunits and AKT pathways [5, 8]. This required us to perform a more in-depth investigation of the underlying mechanisms of GPR34/PI3K in colorectal cancer cell proliferation.

The pathogenesis involves mutations or overexpression of genes encoding proteins including GPCRs and PI3K subunits, which are involved in the regulation of cell survival and proliferation [10–13]. In addition, PI3K subunit overexpression (PI3KCB) and mutations (PI3KCA, PTEN), GPCR overexpression, and/or activation by a wide array of different ligands might effectively and finely control the cell growth and proliferation induced through the PI3K/AKT pathway [10]. Iida et al. proved that lysophosphatidylserine (LysoPS) stimulates chemotactic migration of colorectal cancer cells through the GPR34 and PI3K/Akt pathways in vitro [4]*.*

In this study, we investigated the effects of GPR34 knockdown on proliferation of LS174T colon cancer cells, as well as PI3K subunits/AKT and ERK expression, with the aim of evaluating whether the expression of GPR34 may regulate the growth and proliferation of colon cancer cells, and exploring the details of the underlying mechanisms. Moreover, mRNA-seq datasets from TCGA using Kaplan–Meier survival analysis, the subjects with full clinical data were collected to predict the prognostic implications associated with GPR34 expression. Last, we evaluated the *GPR34* mRNA level of 34 CRC tumor tissue using RT-PCR.

## Materials and methods

### TCGA dataset processing and screening for differentially expressed GPR34

Genome-wide mRNA-seq expression data and clinical materials from CRC patients were obtained from the TCGA website (https://portal.gdc.cance r.gov/). 208 CRC subjects with full clinical information were taken out to perform the Kaplan–Meier survival analysis [14]. The clinical pathological details of the cases are listed in Table 1.

**Table 1:** The Clinicopathological parameters of 208 cases from TCGA database

Clinicopathological parameters	Number of cases	Percentage
Total	208	100
*Age*		
< 65	101	48.6
≥ 65	107	51.4
*Sex*		
Male	112	53.8
Female	96	46.2
*BMI grade*		
0	4	1.9
I	63	30.3
II	80	38.5
III	36	17.3
IV	15	7.2
V	10	4.8
*TNM stage*		
I	30	14.4
II	79	38.0
III	74	35.6
IV	25	12.0
*Tumor stage*		
T1 + T2	33	15.9
T3 + T4	175	84.1
*Lymphovascular invasion*		
Negative	111	53.4
Positive	97	46.6
*Metastases*		
Negative	164	78.8
Positive	44	21.2

### Patient characteristics and sample selection

The tumor tissue and adjacent tissues from 34 CRC patients were collected from Peking University People’s Hospital. Cases with a confirmed diagnosis of colon carcinoma based on histopathology of resected material were included. The clinical pathological details of the cases are listed in Supplementary Table 2. The mRNA expression level of GPR34 in tumor tissues and adjacent normal tissues were then compared. This research project was approved by the Research Ethics Committee of Peking University People’s Hospital (Approval No: 2019PHB028-01) and informed consent forms were signed by all of the subjects prior to participation.

### RNA isolation and complementary DNA preparation

For each CRC patient, a single core tumor biopsy and adjacent tissue greater than 5 cm were taken out. Total RNA was isolated with QIAGEN miRNeasy Mini kit (QIAGEN, Germany) according to the manufacturer’s instructions. Total RNA was reverse transcribed into complementary DNA (cDNA) using PrimeScript™ RT Reagent kit (TAKALA, Japan) according to the manufacturer’s instructions. In brief, each 10 μL reaction mix contained 500 ng of RNA, 5 × master mix and reverse transcriptase. The samples were reverse transcribed for 15 min at 37 ℃ followed by enzyme inactivation for 5 min at 85 ℃.

### Reverse transcription-PCR for GPR34 transcription

The RT-PCR reaction was carried out. Briefly, each 20 μL reaction mixture contained 100 ng of cDNA, and performed using SYBR Green Realtime PCR Master Mix (TOYOBO, Japan). cDNA from the same samples was assayed by RT-PCR in triplicate. Relative quantification was done using GAPDH as an endogenous housekeeping transcription control (forward primer 5′-AATGAAGGGGTCATTGATGG-3′ reverse primer 5′-AAGGTGAAGGTCGGAGTCAA-3′) [15]. Human GPR34 primers were described as previously mentioned [8] (forward primer 5′-CTCCCACAGAATGCGCTTTAT-3′ reverse primer 5′-CAACCAGTCCCACGATGAAAA-3′).

### Cell culture and transfections

LS174T cell line from a women's adenocarcinoma of the colon, was obtained from Cell bank of National Collection of Authenticated Cell Cultures. LS174T was grown in DMEM (HyClone, Beijing, China) containing 10% fetal bovine serum (FBS) (Sigma, St. Louis, MO, USA), and 100 units/mL penicillin plus 100 μg/mL streptomycin (Invitrogen, Beijing, China) in a humidified atmosphere of 5% CO_2_, 95% air at 37 °C. Three MSCV-based pSM2 retroviral vectors containing cDNAs encoding short hairpin RNAs (shRNAs) that were targeted GPR34 were purchased from Open Biosystems (Huntsville, AL, USA). An effective sequence for GPR34 knockdown through targeting by shRNA refers to our previous study [8]. After transfection with the two vectors [control (LS174T-empty vector), GPR34-knockdown (LS174T-GPR34-shRNA)], stable cell-lines were used by resistance to puromycin (2 µg/ml).

### Western immunoblot

Western immunoblot analyses were performed as our previous studies [5, 8, 9]. We used the following antibodies: β-actin (1:2000 dilution, mouse, #HRP-60008, Proteintech), GPR34 (1:2000 dilution, mouse, #H00002857-B01P, Abnova), PI3K Ab Sampler Kit (1:1000 dilution, Rabbit, #9655, CST), P-Akt pathway Sampler Kit (1:1000 dilution, Rabbit, #9916, CST), p-ERK (1:2000 dilution, Rabbit, #4370, CST), ERK (1:1000 dilution, Rabbit, #4695,CST) and PTEN and PDK1 Antibody Sampler Kit (1:1000 dilution, Rabbit, #9652, CST). Total protein (150 μg each) samples were separated on SDS-PAGE gels, transferred to a nitrocellulose membrane, and immunoblotted with appropriate antibodies. Signals were detected using a SuperSignal West Pico Trial Kit (Thermo Scientific, Rockford, IL). Images were acquired using an ImageQuant 350 system (GE HEALTHCARE, USA) and analyzed using ImageJ software (NIH, USA).

### Cell proliferation

Cell proliferation assays were performed with a Cell Counting Kit-8 (CCK-8; Dojindo, Rockville, MD) [16]. 2.5 × 10^4^ of LS174T cell lines expressing GPR34-shRNA, or control (empty vector), were seeded into each well of a 96-well plate. At time-points of 0 h, 24 h, 48 h and 72 h, culture medium was exchanged for 10 μL CCK-8 and 100 μL DMEM, and 96-well plate were incubated for an additional 2 h. The absorbance values at 450 nm in the experimental wells relative to the initial values were calculated to indicate cell growth. The assay was repeated in replicates of three wells per time point. When they reached exponential growth, the transfected LS174T cells were cultured in the presence or absence of LysoPS (10 μM, 20 μM, 40 μM, 80 μM) [4], or GPR34 monoclonal antibody (Mab) (0.2 μg/ml), or PI3K inhibitors 100 nM A66 [17], 100 nM TGX-221 [18], and/or 3 μM Ly290004 [18], all purchased from Selleck Co. USA, for 72 h, repeated in replicates of three wells.

### Soft agar assay

The LS174T-vector and LS174T-GPR34-shRNA cells were seeded at 2 × 10^5^ into each well of a 6-well plate on 0.7% agarose on top of a 1.2% agarose base supplemented with complete medium, which included 10% Fetal Bovine Serum (FBS). Cells were maintained for about 4 weeks [14]. Pictures were taken by a digital camera attached to the microscope and used to count the number of colonies.

### Fura-2 AM calcium assay

Calcium imaging is a common technique that is useful for measuring calcium signals in cultured cells. One of the most common calcium indicators is Fura-2, which has an emission peak at 505 nM and changes its excitation peak from 340 to 380 nm in response to calcium binding. The LS174T cells were cultured in the presence of 20 μM LysoPS for 48 h. The assay was performed in the use of Fura-2 to measure intracellular calcium elevations in LS174T cells. The protocol was described in a previous study [19].

### Animal model

The LS174T-vector, and LS174T-GPR34-shRNA cells were utilized for establishing subcutaneous xenograft tumors. Nude mice aged 8 weeks were purchased from Vital River (Vital River laboratories, Beijing, China) and subcutaneously injected with LS174T, or LS174T-GPR34-shRNA at 2 × 10^6^ cells/mouse. The size of tumors was measured and growth of mice was observed. After 30 days, animals were sacrificed by CO_2_ asphyxiation and were examined for tumors. After weighing the whole body of each mouse, xenograft tumors were excised and weighed. Tumor/body ratio was used as the basis for further analysis. The Institutional Animal Review Committee at the Peking University People’s Hospital approved all protocols used in this study.

### Statistical analysis

SPSS version 22.0 software (SPSS, Chicago, IL, USA) and Graphpad Prism 8.0 (La Jolla, CA, USA) were used for statistical analysis. Data from all quantitative assays were expressed as the mean ± standard deviation (SD) and were analyzed statistically using analysis of variance, two-sided Student’s t test or chi-square test. The follow-up period was computed based on the Kaplan–Meier method using X-tile 3.6.1 (Yale school of medicine). P < 0.05 was considered as statistically significant.

## Results

### Survival analysis for TCGA datasets and GPR34 expression for RT-PCR assay

**Fig. 1:**
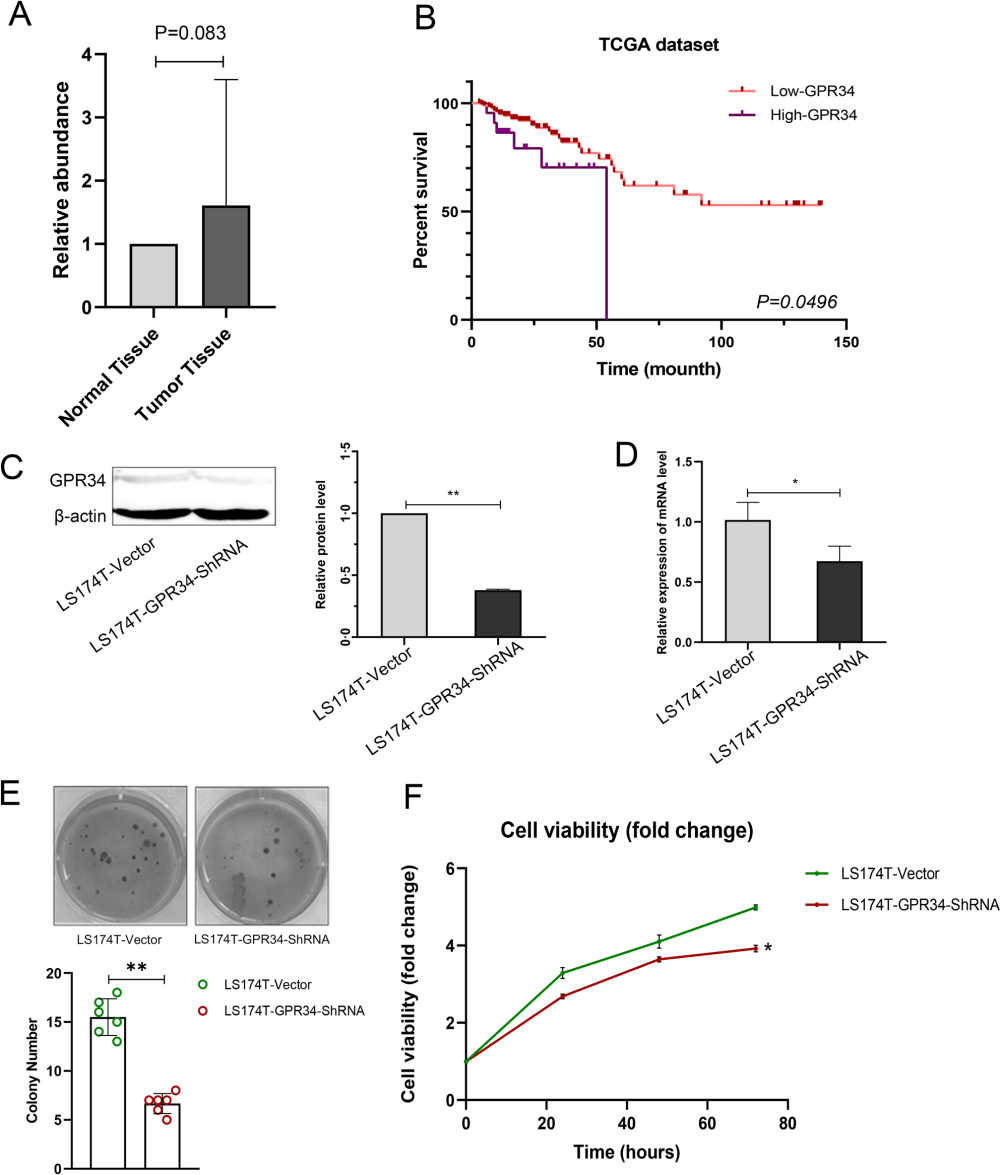
GPR34 expression confirmed by RT-PCR of 34 CRC patients and the Kaplan–Meier survival analysis of 208 CRC patients in TCGA datasets, and Knockdown of GPR34 inhibit the proliferation and colony formation of LS174T cells in vitro.** a** GPR34 expression in tumor samples and adjacent normal tissues by RT-PCR. **b** Kaplan–Meier survival curves of GPR34 related to overall survival (OS). GPR34 was positively correlated with OS. The red lines represent the subjects with low GPR34 low expression, and the purple lines represent the subjects with high expression.** c** Effect of GPR34 knockdown by GPR34-shRNA on protein levels. Image J software analysis. Data represent mean ± SD of triplicate experiments. ** P < 0.01. **d** Effect of GPR34 knockdown induced by GPR34-shRNA on mRNA levels. Total RNAs from the indicated cell lines were isolated and the cDNAs were synthesized. Real-time quantitative PCR was performed to determine *GPR34* mRNA levels, which are expressed as the levels relative to that of β-actin. Data represent mean ± SD of triplicate experiments. *P < 0.05. **e** A soft agar assay showed that GPR34 knockdown impaired LS174T colony formation in vitro. The colony number is shown on the vertical axis as the mean ± SD of triplicate wells. Circles represent plates treated with LS174T-Vector cell or LS174-GPR34-ShRNA cell lines. **P < 0.01. **f** Inhibition of LS174T growth and proliferation by GPR34 knockdown. LS174T-vector cells and LS174T cells transduced with a GPR34 specific shRNA were grown in vitro for the indicated time. A cell counting kit-8 assay showed that GPR34 knockdown significantly impair the proliferative activities of LS174T cells in vitro. The cell viability (fold change) is shown on the vertical axis as the mean ± SD of triplicate wells (2-way ANOVA, **P < 0.01 LS174T-vector vs., LS174-GPR34-ShRNA, n = 3)

The mRNA-seq expression datasets collected from the TCGA database contained 208 patients diagnosed with colorectal cancer, and consisted of 112 men and 96 women. The age at CRC diagnosis ranged from 31 to 90 years (Table 1). Kaplan–Meier survival analysis revealed that in TCGA dataset, patients with high expressed GPR34 showed poor overall survival compared to those with low GPR34 expression, with statistically significant (P = 0.049) (Fig. 1b).

The detailed clinical and pathological characteristics of 34 CRC patients from Peking University People’s Hospital are shown in Supplementary Table 2. GPR34 expression showed a tendency of higher expression in tumor tissues, compared with adjacent normal tissues (P = 0.083) (Fig. 1a). However, *GPR34* mRNA level was not significantly correlated with various clinicopathological GC parameters, including TNM stage, tumor stage, Lymphovascular invasion and distal metastasis, etc. (Supplementary Table 1).

### Construction of stable GPR34 knockdown with LS174T cell models

It has previously shown that GPR34 is upregulated in colon cancer tissues and cell lines [20]. To determine the role of GPR34 in growth and proliferation of LS174T, stable cell colonies were selected by puromycin and tested for the effects of shRNA on GPR34 expression. As shown in Fig. 1d, expression of shRNA in LS174T cells resulted in a 35% decrease in *GPR34* mRNA, compared with the LS174T-vector cells. By Western blot analysis, the expression of GPR34 was significantly reduced in LS174T-GPR34-shRNA cells, as compared with LS174T-vector cells (Fig. 1c).

### Knockdown of GPR34 inhibit the proliferation and colony formation of LS174T cells in vitro

Given that GPR34 is a key regulator of growth and proliferation, we tested whether the GPR34 knockdown could affect abnormal proliferative functions. A soft agar assay also showed that GPR34 knockdown impaired LS174T colony formation in vitro (Fig. 1e). A cell counting kit-8 assay showed that GPR34 knockdown significantly impaired the colony formation of LS174T cells in vitro (Fig. 1f). Expression of GPR34 shRNA in LS174T cells resulted in significant reduction in proliferation.

### Xenograft tumor growth is impaired in vivo by GPR34 knockdown in LS174T cells

**Fig. 2:**
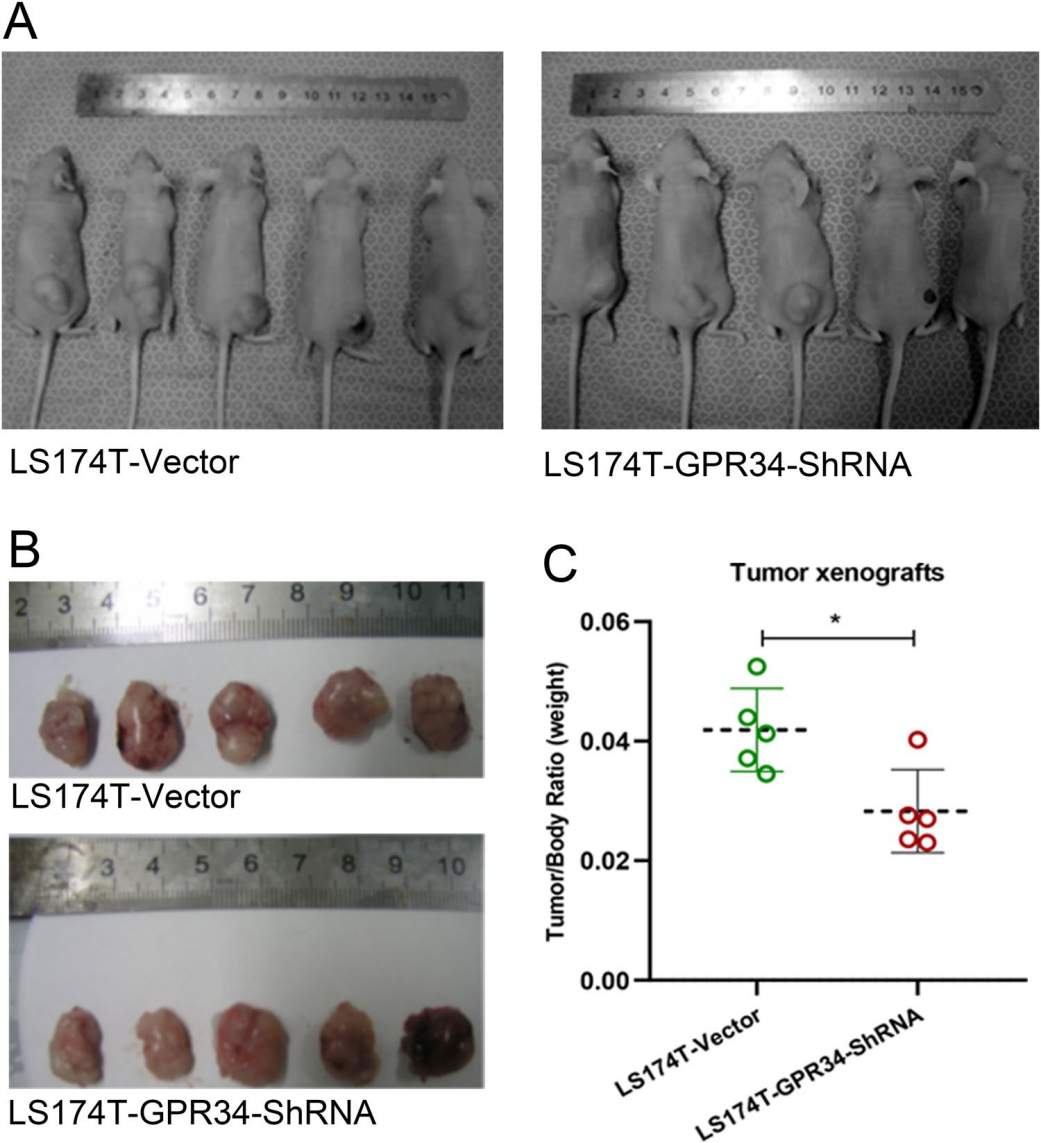
Xenograft tumor growth is impaired in vivo by knockdown of GPR34 in LS174T cells. **a** Tumors established in null BALB/c mice by subcutaneous injection of 2 × 10^6^ LS174T-GPR34-shRNA, LS174T-vector cells. Thirty days later, subcutaneous injection of LS174T cells resulted in tumor formation in all 15 mice (tumor formation rate 100%). **b** Tumors were excised and weighed. **c** The tumor/body ratio (weight) is shown on the vertical axis as the mean ± SD of 5 mice (*P < 0.05, LS174T-vector vs. LS174-GPR34-ShRNA, n = 5/group)

To test whether GPR34 knockdown in LS174T cells affects xenograft tumor growth in vivo, stable LS174T-GPR34-ShRNA, and LS174T-vector cells were injected subcutaneously into nude mice. The recipient mice were followed for the development of xenograft tumors and sacrificed 30 days after injection (Fig. 2a). The xenograft tumors were isolated and weighed (Fig. 2b). The ratio of xenograft weight and whole body weight of mice was used to evaluate the effects of GPR34 expression on the growth of xenograft tumors (Fig. 2c). Consistent with the in vitro results, GPR34 knockdown in LS174T cells inhibited xenograft tumor growth in vivo, compared with LS174T-vector injected mice.

### LysoPS stimulates while GPR34 monoclonal antibody (GPR34 Mab) inhibits the growth and proliferation of LS174T-vector cells

**Fig. 3:**
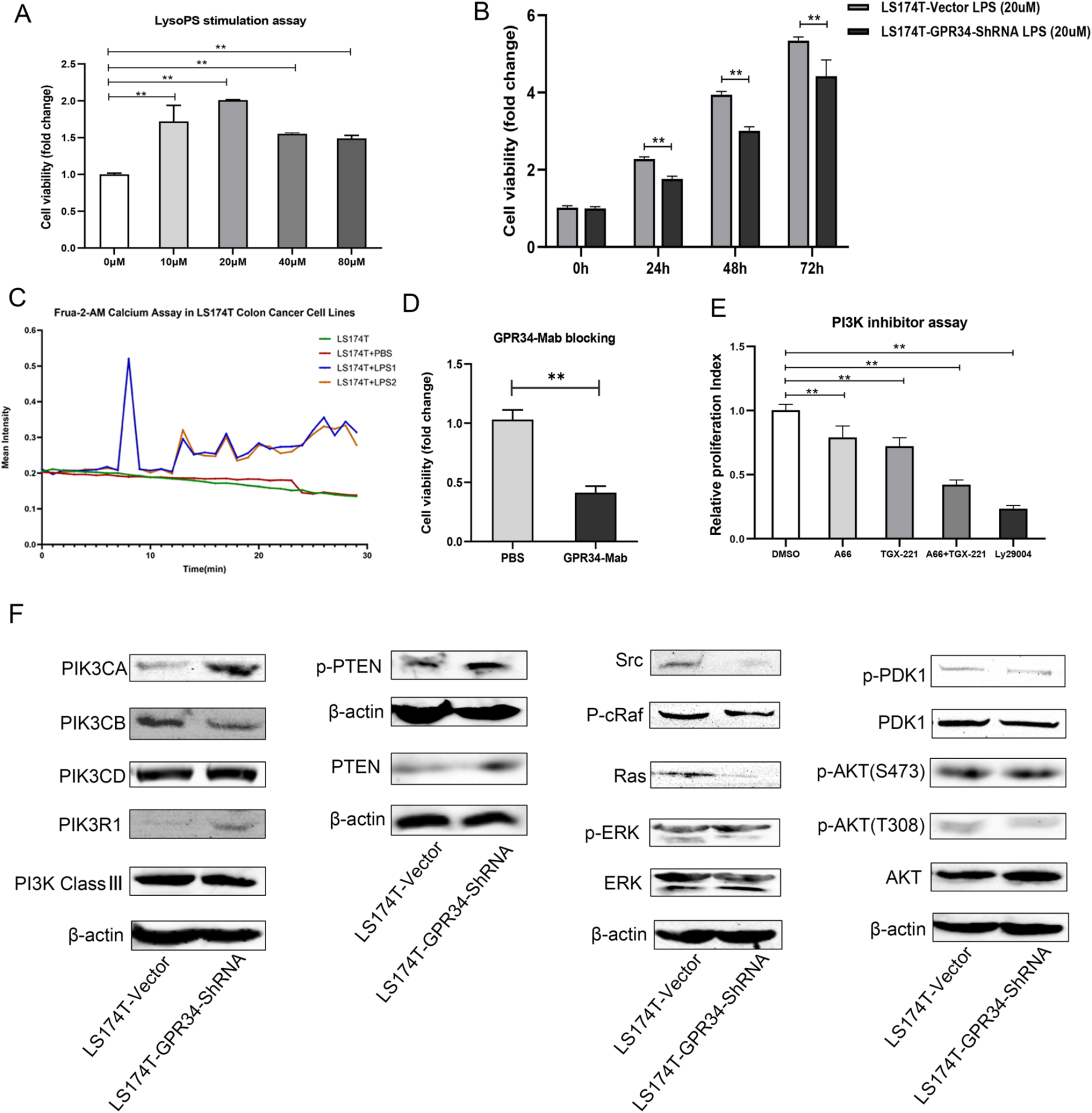
The stimulatory effect of LysoPS, GPR34-Mab and PI3K inhibitor assay on the proliferation of LS174T cells. **a** Proliferation assay showing a significant stimulatory effect of lysoPS on LS174T-vector cells. Cancer cells were treated with various concentrations of lysoPS for 72 h. The cell viability (fold change) is shown on the vertical axis as the mean ± SD (1-way ANOVA, **P < 0.01 vs. control, n = 3). **b** Proliferation assay showing a significant stimulatory effect of lysoPS with 20 μM on LS174T-GPR34-ShRNA cells or LS174T-vector cells at 0 h, 24 h, 48 h, 72 h. The cell viability (fold change) is shown on the vertical axis as the mean ± SD (2-way ANOVA, **P < 0.01 vs. control, n = 3). **c** Frua-2-AM Calcium Assay in LS174T cell lines. LS174T cell lines was treated by lysoPS and PBS for 72 h, separately. The cell intensity was monitored within 30 min every 15 s. **d** Proliferation assay showing a significant inhibitory effect of GPR34-Mab blocking on LS174T-vector cells. Cancer cells were treated with 0.2 μg/mL of GPR34-Mab for 72 h and cell viability was analyzed. The cell viability (fold change) is shown on the vertical axis as the mean ± SD (**P < 0.01 vs. control, n = 3). **e** A66 (PI3KCA-specific inhibitor), TGX-221 (PI3KCB-specific inhibitor), and LY29004 (PI3K universal inhibitor) blocking assay. After treatment with A66 (100 nM), TGX-221 (100 nM), A66 + TGX-221 (each 100 nM), or LY29004 (3 μM), a cell viability assay using cck-8 was performed. Cell viability (fold change) of these cells is shown on the vertical axis as mean ± SD of triplicate wells. As indicated, all three inhibitors, A66, TGX-221, and LY290045, were found to inhibit the growth and proliferation of LS174T-vector to various degrees compared with DMSO treatment. The cell viability (fold change) is shown on the vertical axis as the mean ± SD (**P < 0.01 vs. DMSO, n = 3).** f** GPR34 controls the proliferation of LS174T cells through different pathways based on distinct PI3K subunits. PI3K/PTEN, GPR34/PI3K/ERK, GPR34/PI3K/PDK1/AKT and represent potential pathways which regulate the growth and proliferation of cancer cells. Basal expression of PI3KCB (p110β), and PI3K Class III, compared with LS174T-vector cells. PI3KCB was found to be downregulated in LS174T-GPR34-shRNA. Both phosphorylated and non-phosphorylated PTEN were constitutively (significantly) upregulated. Low levels of p-Src, pc-Raf, Ras and p-ERK expression were observed in LS174T-GPR34-shRNA cells, and lower expression levels of p-PDK1 and p-AKT(T308) were detected in LS174T-GPR34-shRNA cells

In order to further determine the effects of GPR34 on the growth and proliferation of LS174T cells, exogenous LysoPS (optimized, 20 μM, Fig. 3a) proved as a stimulation for the migration of colorectal cancer cells through GPR34 and PI3K/Akt pathway, was added into the complete DMEM media. In contrast to the results of Iida et al. [4], our results showed a significant stimulatory effect of 20 μM LysoPS on LS174T-vector cell proliferation, compared with LS174T-GPR34-ShRNA cell (Fig. 3b). We also found that the addition of GPR34 monoclonal antibody (GPR34 Mab) (0.2 μg/ml, Fig. 3d) resulted in a significant inhibitory effect on LS174T-vector cell proliferation. Taken together with the results of GPR34 knockdown, evidence indicates that GPR34 plays an important role in regulating the growth and proliferation of LS174T cells, using a complex underlying mechanism.

PI3K subunits have been shown to be involved in the growth and proliferation of various cancer cells [10]. To determine whether PI3K subunits control the growth and proliferation of LS174T cells, LS174T-vector cells were exposed to either 100 nM A66 (a PI3KCA-specific inhibitor) [17], 100 nM TGX-221 (a PI3KCB-specific inhibitor) [18], A66 + TGX221, or 3 μM LY290004 (an inhibitor of all PI3K subunits) [18], the concentration of the inhibitors was used as previous studies described. As shown in Fig. 3e, the proliferative ability of LS174T-vector cells was inhibited by all of these PI3K inhibitors. The combination of A66 and TGX-221 displayed an additive inhibitory effect on proliferation of LS174T cells, with more inhibition than A66 or TGX-221 alone. This finding indicates that different PI3K subunits are involved in the growth and proliferation of LS174T cells. Furthermore, as shown in Calcium assay (Fig. 3c), 20 μM LysoPS was proved play a role on the growth of LS174T-vector cell.

### GPR34 regulates the growth and proliferation of LS174T through differential expression of PI3K subunits

**Fig. 4:**
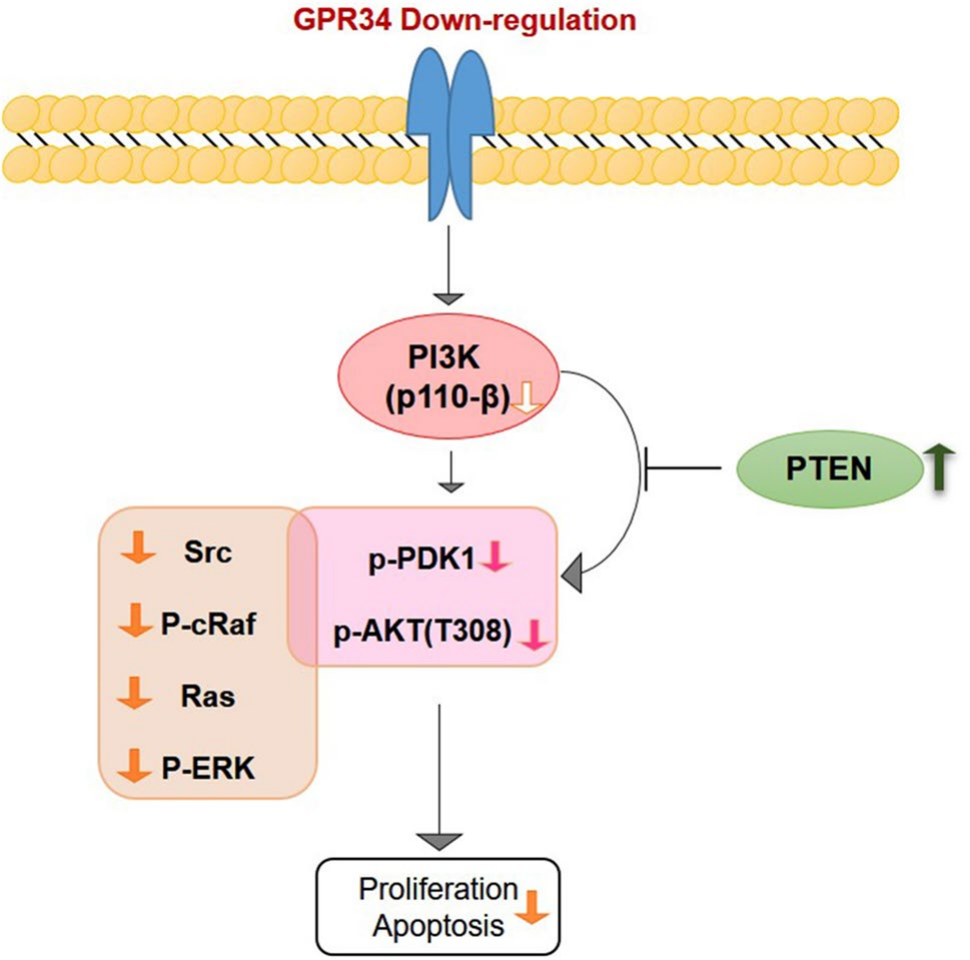
GPR34 signal transduction. GPR34 is a G-protein coupled receptor that activates classical signaling cascades downstream this G-protein family. This includes activation of GPR34/PI3K/ERK and R34/PI3K/PDK1/AKT pathway, which is critical for cell proliferation

It has been widely accepted that PI3K/AKT and/or ERK are critical players that act the downstream of G-protein coupled receptors to stimulate cell growth and survival [5]. We have previously shown that GPR34 is involved in the proliferation of gastric cancer and leukemic cells through PI3K/AKT and/or ERK pathways [5, 8, 9]. To determine the mechanism by which GPR34 contributes to growth and proliferation of LS174T cells, we examined the effect of GPR34 knockdown on activation of PI3K/AKT and ERK signaling pathways. Firstly, as Fig. 3f shown, among the PI3K class I subunits, PI3KCA (p110α) and PI3KR1 (p85) are significantly upregulated in LS174T-GPR34-shRNA cells, while PI3KCB (p110β) is significantly downregulated in LS174T-GPR34-shRNA cells. There were no changes in PI3KCD (p110δ) or PI3K Class III expression in all three tested cells. Secondly, we found that both non-phosphorylated and phosphorylated PTEN (phosphatase and tensin homolog) were constitutively upregulated in LS174T-GPR34-shRNA compared with LS174T-vector. Thirdly, we checked the expression of phosphorylation and non-phosphorylation of PDK1/AKT and ERK), and found that expression of phosphorylation PDK1 and (p-AKT (T380)) were downregulated in LS174T-GPR34-shRNA compared with LS174T-vector, except for p-AKT (S473). Last, the expression of Src, p-cRaf, Ras, and phosphorylated ERK were reduced in LS174T-GPR34-shRNA. Interestingly, phosphorylated PDK1, and Ras, which belong to the PI3K/PDK1/AKT and PI3K/Ras/Src/Raf/ERK pathways respectively, were found to be expressed as the same as the expression of PI3KCB in these two cell lines. GPR34 activates classical signaling cascades downstream of this G-protein family. This includes activation of GPR34/PI3K/ERK and R34/PI3K/PDK1/AKT pathway, which is critical for cell proliferation (Fig. 4).

## Discussion

In this work, the expression of GPR34 was shown significantly higher in CRC tumor samples. We then examined the potential relationships between the mRNA levels of GPR34 in CRC and its clinical parameters. We found no other correlations between *GPR34* mRNA levels and the clinical parameters. However, the increased expression of GPR34 is associated with poor prognosis of CRC in TCGA dataset. These results suggest GPR34 has a high tumor correlation and play important roles in the colon tumorigenesis and progression. Thus, illustrating the underlying molecular mechanism of the occurrence and development of CRC is extremely important. To further explore the underlying mechanism, the relationship between GPR34 expression and proliferation and xenograft tumor growth of colon cancer cells, as well as the potential mechanism underlying this relationship were achieved through a combination of GPR34 functional analysis, ligand stimulation, specific-antibody blocking, and signal pathway analysis.

It was reported that GPR34 was upregulated in colon cancer cell lines and tissues [20], and might act as a potential proto-oncogene involved in the development and progression of colon cancer. Since GPR34 expression was increased in LS174T cells, we studied the role of this signaling molecule in the context of proliferative and colony formation properties in vitro and xenograft tumor growth in vivo. Using stable GPR34 knockdown LS174T cell models, we found that GPR34 loss-of-function resulted in impaired proliferation, colony formation, and xenograft tumor growth (Fig. 1 and Fig. 2). Furthermore, we treated LS174T cells with the GPR34-specific ligand LysoPS (agonist) [21] and GPR34-specific Mab, and found that 20 μM LysoPS and 0.2 μg/mL GPR34-Mab (Fig. 3) had strongly stimulatory and inhibitory effects on the proliferation of LS174T cells respectively, indicating that GPR34 expression is involved in the proliferation and growth of LS174T cells. Then, Fura-2 AM Calcium assay was performed, indicating that the stimulated GPR34 may mediate proliferation of LS174T by Calcium ion, which was reported to regulate the PI3K-Akt pathway [22].

By regulating PI3K/AKT and/or PI3K/ERK activity, GPCRs are able to bring about the resulting biochemical and physiological changes, such as cellular proliferation, apoptosis and survival [20, 23]. We have previously shown that GPR34/PI3K/AKT and GPR34/PI3K/ERK play important roles in proliferation, apoptosis, and migration in gastric cancer [5, 8] and BCR/ABL-positive leukemia. In gastric cancer cells, GPR34 knockdown downregulated not only the expression of p-PI3KR1 in NCI-N87 cells, but also the expression of PI3KCD (p110δ) in HGC-27 cells [5, 8]. In mutant BCR/ABL-transformed Ba/F3 cells (p185YR), GPR34 knockdown also decreased the expression of PI3KCD, but increased the expression of non-phosphorylated and phosphorylated PI3KR1, as well as non-phosphorylated PTEN. The main function of PTEN is to block the PI3K pathway by dephosphorylating phosphatidylinositol (PI) 3,4,5-triphosphate to PI-4,5-bisphosphate thus counteracting PI3K function [24, 25]. All these findings indicate that GPR34 exerts a cancer cell specific effect on the expression of PI3K subunits through upregulating PTEN. Given the importance of GPR34-mediated signaling in regulating proliferation and tumorigenesis of LS174T cells, it is conceivable that GPR34/PI3K subunits/AKT and GPR34/PI3K subunits/ERK serve as two very important pathways in regulating the proliferation and growth of LS174T [26].

In line with hypothesis of GPR34 knockdown on regulation of PI3K/AKT and ERK signaling pathways, we examined LS174T cell-specific expression profiles of class I PI3K subunits and their downstream effectors under GPR34 knockdown condition. We found significant expression of PI3K subunits (p110β subunit), phosphorylated and non-phosphorylated PTEN, phosphorylated PDK1, AKT, Src, Raf, and ERK, in LS174T-GPR34-shRNA cell. Compared with LS174T-vector cells, PI3K subunit β, phosphorylated AKT (T380), and Ras were obviously downregulated in LS174T-GPR34-shRNA cell (Fig. 3). Non-phosphorylated and phosphorylated PTEN were upregulated in LS174T-GPR34-shRNA cell (Fig. 3f), while phosphorylated PDK1 and p-AKT(T380), Src, Raf, and ERK were all downregulated in LS174T-GPR34-shRNA cell line. These observations suggest that GPR34 as an independent factor regulates the changes in PI3K subunits, PTEN, PDK1/AKT, and Src/Raf/ERK molecules. In LS174T-GPR34-shRNA cells, downregulation of PI3KCB and p-AKT (T380), might play a key role in inhibiting LS174T cellular proliferation.

It was essential to determine whether PI3K subunits, especially PI3KCB, are key “controllers”. Experiments using the inhibitors A66 (100 nM), TGX221 (100 nM), and LY290004 (3 µM) were performed to confirm the roles of PI3K subunits in the proliferation of LS174T cells. As Fig. 3e indicates, both A66 and TGX-221 inhibit the proliferation of LS174T cells, and A66 plus TGX-221 produced an inhibitory effect on the proliferation of LS174T cells. Taken together, these observations support the hypothesis that GPR34 knockdown might control the growth and proliferation of LS174T cells through PI3K subunits, especially PI3KCB.

GPR34 upregulation has been detected in various tumor tissues, including melanoma [6], human MZL cells [1, 2], leukemia [9], stomach [5, 8], and cervical cancer [7]. Overexpression of GPR34 in lymphoma resulted in phosphorylation of ERK, induced NF-κB-mediated gene transcription, and increased cell proliferation [1]. We have showed that GPR34 might activate PI3K/AKT signaling pathways by regulating the balance between p110–p85 and p85–PTEN activities to induce IL-3-independent antiapoptosis, proliferation, and survival in Ba/F3 cells transformed by p185YR [9]. Nowadays, we first showed that GPR34 might finely control the growth and proliferation of LS174T through different pathways based on PI3K subunits, especially PI3KCB. These results also suggest that LS174T GPR34 knockdown model systems might serve as useful tools in the analysis of molecular mechanisms underlying colon cancer progression. This could lead to the construction of more precise therapeutic strategies. It also indicates the possibility of important roles for PI3K/AKT and ERK pathways in radiotherapy resistance [27] and tissue repair [28].

Combined with previous data from gastric cancer and leukemic cells, our present data from LS174T cells indicates the need for additional, related assays to confirm the complex roles and underlying mechanism of GPR34 in the progression and development of colorectal cancer and other cancers. Considering the complexity and interconnectedness of cellular signaling networks, such studies are essential in order to provide better tools for stratification of patients into smaller subgroups to which specific targeted therapy may be administered. In addition, they would provide invaluable clues as to how components in this network could be targeted by different strategies [10, 29, 30]. Our study is limited by the lack of more cell CRC-related cell lines to validate the effects of GPR34 in the proliferation of different cell lines. Further studies will be required to additional cancer cell lines of the colon to define the role of GPR34.

## Supplementary Information

Below is the link to the electronic supplementary material.

Supplementary file1 (DOCX 14 kb)

Supplementary file2 (DOCX 13 kb)

## Data Availability

The datasets generated during and/or analysed during the current study are not publicly available due to privacy or ethical restrictions but are available from the corresponding author on reasonable request.
