# Supplementary material for: G-protein coupled receptor 34 regulates the proliferation and growth of LS174T cells through differential expression of PI3K subunits and PTEN
Source: Mol Biol Rep. 2022 Jan 8;49(4):2629–39. doi: 10.1007/s11033-021-07068-4 (PMC8924081; doi:10.1007/s11033-021-07068-4)
Supplement: Supplementary file 1 — Supplementary file1 (DOCX 14 kb) [file 11033_2021_7068_MOESM1_ESM.docx]

Supplementary Table 1. Correlation between the clinicopathological parameters and GPR34 expression of 34 colon cancer patients.

| Clinicopathological  parameters | Numeber of cases | GPR34  expression level | | Pearson  Chi-square | | *P* value | |  |
| --- | --- | --- | --- | --- | --- | --- | --- | --- |
|  |  | Low | High |  |  |  |  |  |
| Total | 34 | 17 | 17 | |  | |  | |
| Age |  |  |  | | 0.654 | | 0.686 | |
| <65 | 8 | 5 | 3 | |  | |  | |
| ≥65 | 26 | 12 | 14 | |  | |  | |
| Sex |  |  |  | | 0.486 | | 0.486 | |
| Male | 20 | 11 | 9 | |  | |  | |
| Female | 14 | 6 | 8 | |  | |  | |
| TNM stage |  |  |  | | 0.118 | | 0.732 | |
| 0-II | 17 | 8 | 9 | |  | |  | |
| III-IV | 17 | 9 | 8 | |  | |  | |
| Tumor stage |  |  |  | | 0 | | 1 | |
| T1+T2 | 6 | 3 | 3 | |  | |  | |
| T3+T4 | 28 | 14 | 14 | |  | |  | |
| Lymphovascular invasion |  |  |  | | 0.125 | | 0.724 | |
| Negative | 21 | 11 | 10 | |  | |  | |
| Positive | 13 | 6 | 7 | |  | |  | |
| Metastases |  |  |  | | 0 | | 1 | |
| Negative | 27 | 14 | 13 | |  | |  | |
| Positive | 7 | 3 | 4 | |  | |  | |
