# Supplementary material for: G-protein coupled receptor 34 regulates the proliferation and growth of LS174T cells through differential expression of PI3K subunits and PTEN
Source: Mol Biol Rep. 2022 Jan 8;49(4):2629–39. doi: 10.1007/s11033-021-07068-4 (PMC8924081; doi:10.1007/s11033-021-07068-4)
Supplement: Supplementary file 2 — Supplementary file2 (DOCX 13 kb) [file 11033_2021_7068_MOESM2_ESM.docx]

Supplementary Table 2. The Clinicopathological parameters of 34 colon cancer patients

| Clinicopathological  parameters | Numeber of cases | Percentage |
| --- | --- | --- |
| Total | 34 | 100% |
| Age |  |  |
| <65 | 8 | 23.5% |
| ≥65 | 26 | 76.5% |
| Sex |  |  |
| Male | 20 | 58.8% |
| Female | 14 | 41.2% |
| TNM stage |  |  |
| I | 6 | 17.6% |
| II | 11 | 32.4% |
| III | 10 | 29.4% |
| IV | 7 | 20.6% |
| Tumor stage |  |  |
| T1+T2 | 6 | 17.6% |
| T3+T4 | 28 | 82.4% |
| Lymphovascular invasion |  |  |
| Negative | 21 | 61.8% |
| Positive | 13 | 38.2% |
| Metastases |  |  |
| Negative | 27 | 79.4% |
| Positive | 7 | 20.6% |
